# Deep learning for fast low-field MRI acquisitions

**DOI:** 10.1038/s41598-022-14039-7

**Published:** 2022-07-06

**Authors:** Reina Ayde, Tobias Senft, Najat Salameh, Mathieu Sarracanie

**Affiliations:** Center for Adaptable MRI Technology (AMT Center), Department of Biomedical Engineering, University of Basel, Allschwil, Switzerland

**Keywords:** Biomedical engineering, Magnetic resonance imaging, Computer science

## Abstract

Low-field (LF) MRI research currently gains momentum from its potential to offer reduced costs and reduced footprints translating into wider accessibility. However, the impeded signal-to-noise ratio inherent to lower magnetic fields can have a significant impact on acquisition times that challenges LF clinical relevance. Undersampling is an effective way to speed up acquisitions in MRI, and recent work has shown encouraging results when combined with deep learning (DL). Yet, training DL models generally requires large databases that are not yet available at LF regimes. Here, we demonstrate the capability of Residual U-net combined with data augmentation to reconstruct magnitude and phase information of undersampled LF MRI scans at 0.1 T with a limited training dataset (n = 10). The model performance was first evaluated in a retrospective study for different acceleration rates and sampling patterns. Ultimately, the DL approach was validated on prospectively acquired, fivefold undersampled LF data. With varying performances associated to the adopted sampling scheme, our results show that the approach investigated can preserve the global structure and the details sharpness in the reconstructed magnitude and phase images. Overall, promising results could be obtained on acquired LF MR images that may bring this research closer to clinical implementation.

## Introduction

Low-field (LF) magnetic resonance imaging (MRI) has recently regained attention, with multiple initiatives aiming not only to democratize MRI worldwide, but also to complement conventional high-field MRI. Mostly driven by simpler and scalable magnet construction, LF MRI offers increased accessibility from reduced purchasing and maintenance costs, cryogenic-free infrastructure, reduced susceptibility artifacts, higher T1 contrast, and potential for smaller footprint designs^1–4^. Yet, LF MRI suffers from lower sensitivity due to an intrinsically lower nuclear spin polarization. Consequently, the associated lower signal-to-noise ratio (SNR) per unit time and volume almost invariably requires averaging and hence prolonged acquisition times that may restrict its clinical relevance. Technical progress achieved over the past decades in diverse areas of MRI, e.g. power electronics, radio frequency detection, sequence programming, and image processing, have contributed to bring LF back to the surface^1,2^ but further acceleration remains paramount to push the achievable SNR and voxel resolution per unit time, aiming at a broad deployment in radiology departments and beyond.

Regardless of magnetic field strength, MRI requires one to encode the signal originating from ^1^H nuclei to make an image, that may result in long imaging times. *k*-space undersampling strategies have been largely used to accelerate acquisitions, but typically lead to information loss or reconstruction artifacts after Fourier Transform (FT). Restoring the unaltered image becomes then an ill-posed, inverse problem^5^ where regularization methods involving prior knowledge such as the sparsity of MR data in a certain domain (e.g. Wavelet domain) can be leveraged, as done in Compressed Sensing (CS)^6^. Yet, the application of CS in clinical routine is still limited as it requires complex parameter tuning, sometimes very specific to an application or image type, and suffers from long online reconstruction times. An alternative approach to accelerating MR acquisitions is parallel imaging (PI)^7,8^. As opposed to CS, PI is broadly spread and employed in clinical routine. In PI methods, the undersampled *k*-space is acquired using multiple receiver coils and the spatial dependence of their ${B}_{1}^{-}$ field is leveraged to turn the initial ill-posed to a well-posed inverse problem that can be solved by a direct matrix inversion^8^. As noise is severely amplified at high undersampling rates though (characterized by the g-Factor), a limited speed-up rate of fourfold (maximum) is generally observed. Relying on body noise regime dominance to exploit the spatial dependency of coil elements and spatially altered SNR, PI shall however not be indicated to accelerate MRI below a certain magnetic field strength. Indeed, below 5 MHz, sample noise may no longer dominate in the acquisition chain^9^ and such a reconstruction paradigm no longer holds.

Deep Learning (DL) is an emerging field of research that has shown promising results in a variety of domains, including MRI. In fact, DL has provided a new paradigm to solve ill-posed inverse problems, and particularly the reconstruction of undersampled MR acquisitions where superior performance to widespread CS and PI acceleration methods was shown^10–15^. Here, we hypothesize that DL could be a serious means to accelerated LF MRI.

In DL approaches, neural networks made of numerous layers (i.e. referred to as “deep”) are used to learn the complex functions that directly map an input data to a corresponding output. DL models, and especially data-driven approaches with a large number of free parameters require large training set to avoid what is called overfitting^5^. Overfitting occurs when a model learns from a training set which variety is limited, hence failing to generalize to images too different from the training set. Considering such limitation, initiatives were proposed to provide large-scale, open access databases of processed MR images^16–19^ or raw space^20^ to researchers in the MRI community. Interestingly, two aspects in undersampled DL-MR works often seem overlooked, namely prospective validation and phase image reconstruction. Prospective validation is a key step though, to properly assess the performance of proposed methods on undersampled data acquired in real conditions (i.e. not subsampled a posteriori). The integration of complex data inherent to MRI could additionally open new perspectives for the application of DL with phase-contrast MR techniques such as magnetic susceptibility mapping, or further flow and motion encoding. It may also provide more accurate outcomes as MRI data does not limit itself to most used magnitude information. Yet only little research has reported on phase data reconstruction, none of which were validated prospectively^21,22^. Meanwhile, it should be noted that several studies were inherently limited by their model architecture that cannot handle complex input data, or by the nature of the training set constituted solely of magnitude images.

At LF, few attempts have yet been reported that leverage DL for undersampled MRI data reconstruction^23^. Schlemper et al*.* developed a non-uniform variational model to simultaneously reconstruct and recover artifacts from undersampling (3.5-fold) and hardware for a point-of-care scanner (64 mT). In a different work, Koonjoo et al. exploited a model named AUTOMAP to improve SNR at low field, respectively 6.5 mT and 47 mT^24^.

In general, DL approaches face a major challenge at LF: there is no large-scale LF database acquired at the same magnetic field strength. Indeed, the number of scanners/clinical exams at LF are still very limited and there is no consensus as to what field regime is LF MRI. Consequently, LF databases remain private and rather small both on global (worldwide) and laboratory scales. The above-mentioned LF studies relied on an open access conventional (i.e., high-field) MR database from the human connectome project (HCP)^19^ to train and test their model. The latter was pre-processed ad hoc by adding noise^23,24^ and artifacts that would result from patient motion to simulate LF brain data, and account for the limited gradients linearity specific to the system used^23^. Despite such processing, peculiarities such as noise regime and image contrast at LF can be quite different from what is expected at conventional fields (1.5–3 T). Typically, the magnetic properties of biological tissue change at low field, leading to a superior T1 dispersion that implies different soft tissue contrast, and noise in the reception chain may no longer be dominated by the sample but instead by thermal noise from the electronics. Therefore, training with preprocessed conventional field data might very well have a negative impact on reconstruction outcomes. First, this can translate in decreased model performance because of the discrepancy between training and test sets^25^. Second, it might lead to an undesired contrast transfer from conventional field to LF DL reconstructed MR images. Finally, we will note that for both LF DL approaches referenced above, the phase information was not considered as the HCP database contains only magnitude images.

In the proposed work, we explore the capability of a data-driven DL approach to accelerate LF MR acquisitions (here, at 0.1 T) while maintaining both magnitude and phase information. A particular emphasis was brought to tackle challenges associated with small datasets typically encountered at low field. Here, a relatively small dataset (n = 10) of human wrist images was collected and data augmentation was employed to mitigate data scarcity while preventing the deviation between training and testing sets. With data augmentation, the size of a small dataset is artificially expanded based on basic image manipulations (e.g. rotation, translation, cropping, noise injection, etc.) or on deep learning (e.g. generative adversarial networks)^26^. We used 2-channel U-net^27^, one of the state-of-the-art deep learning architectures^11,12,20,28–30^, as an image domain learning model. Its performance was first evaluated on magnitude and phase reconstructed images for three different acceleration rates (R = 3, 4 and 5). Then, in an attempt to preserve high frequencies at the highest acceleration rate (R = 5), the model performance was investigated for undersampling schemes describing different *k*-space coverage. Finally, the proposed approach was validated on both retrospective and acquired, prospective 3D LF data, keeping a keen eye towards clinical transfer.

## Materials and methods

### Low-field data

A total of 10 fully sampled, 3D spoiled gradient echo (GRE) in vivo MR images of the human hand and wrist were acquired at 0.1 T, on a compact, biplanar system using a custom-built transmit/receive coil tuned at F_0_ = 4.256 MHz^31,32^. All data were collected with the following imaging parameters: matrix size = 128 × 115 × 9, voxel size = [1.2 × 1.2 × 6.3] mm^3^, TE/TR = 7.2/31 ms, bandwidth = 17 kHz, flip angle (FA) = 70°, number of averages (NA) = 28 (acquisition time = 14 min 56 s). The dataset was split by randomly choosing 80% (8 sets) for training and 20% (2 sets) for validation. Retrospective and prospective test data consisted each of 6 additional sets of 3D images acquired with the same protocol described above. All MRI experiments were conducted following the local ethics regulations and informed consent was obtained from all subjects. The study was approved by the Ethikkommission Nordwest- und Zentralschweiz (EKNZ) (project-ID 2022-00348).

### Data preprocessing

Each 3D *k*-space was zero-filled to reach 128 × 128 × 9 matrix dimension and fit the square input/output dimensions required by U-net. Even though training with non-square input dimensions (128 × 115) is feasible, we chose to avoid the potential problem of stride and padding size adjustments that could be encountered in this case. Each *k*-space was then inverse Fourier transformed to generate a complex 3D image. To avoid potential overfitting caused by the rather small size of our dataset and improve the model performance, data augmentation was employed. The Keras library^33^ provides a 2D ImageDataGenerator class to facilitate data augmentation implementation. To handle 3D image augmentation, a modified version of Keras class was used^34^. More specifically, for a given 3D complex image, two data augmentation functions were jointly used on the real and imaginary part that include a random selection of one of the following transformations: horizontal and vertical shift (range [0, ± 50 pixels]), horizontal and vertical flip, shear (range [0, 45°]), zoom (range [75%, 1.25%]) and rotation (range [0°, 360°]).

The ‘augmented’ 3D complex image set was then Fourier transformed back to *k*-space domain and retrospectively undersampled on both the first and second phase encode directions (k_y_ and k_z_). Afterwards, data normalization was performed by dividing each 3D *k*-space with its corresponding standard deviation. Pairs of input/output, undersampled and fully sampled complex images were finally obtained following an inverse Fourier transform to go back to the image domain.

### Model architecture and training details

**Figure 1:**
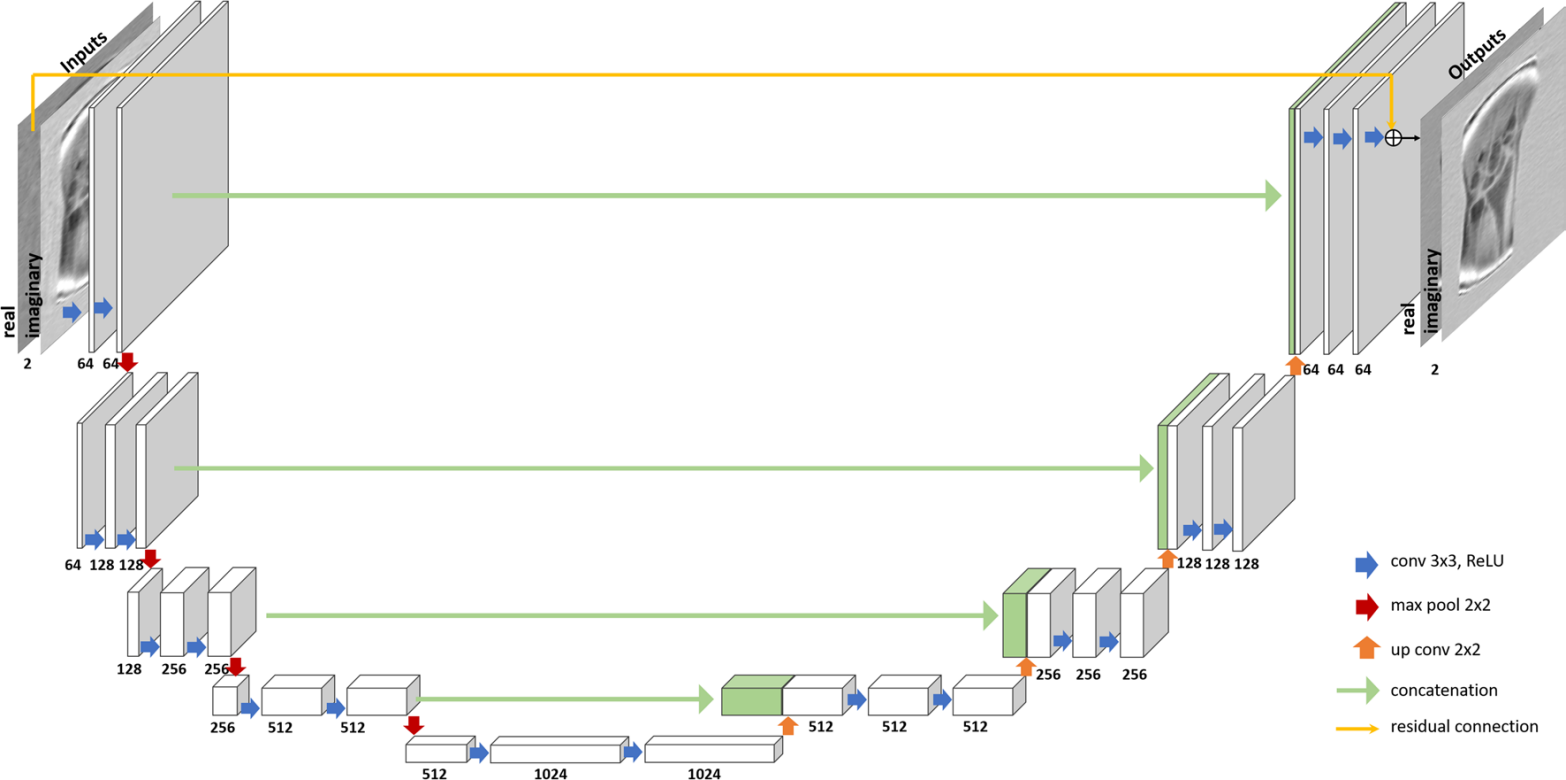
The chosen Residual U-net used to reconstruct LF undersampled data.

In undersampled MRI, the aim is to find an optimal reconstruction function $f:x\to y$, which maps an undersampled image $x$ to a fully sampled image $y$. $f$ can be formulated as follows:

1 $$f=\underset{\mathit{f }}{\mathit{argmin}}\mathcal{L}\left[f\left(x\right)-y\right]$$

with $\mathcal{L}$ being the loss function. $x$ is generated retrospectively using the acquired fully sampled *k*-space: $x= iFT(U(k))$, where $U$ denotes the sampling pattern and $k$ the fully acquired *k*-space. To train our model, the mean squared error (MSE) was used as a loss function. Subsequently, the later formula can be expressed as follows:

2 $$\mathit{f}=\underset{{f }}{\mathit{argmin}}\sum_{1}^{\mathit{N}}{\Vert \mathit{f}\left({\mathit{x}}^{\mathrm{i}}\right)-{\mathit{y}}^{\mathrm{i}}\Vert }^{2}$$

where $i$ indicates the ith sample in the set and $N$ is the total number of samples used to compute the loss. In this work, the deep convolutional neural network architecture U-Net was used as our reconstruction function $f$. U-net consists of an encoding path providing low-level features followed by a decoding path that enables precise localization of these features. Feature localization is further improved thanks to concatenation operations that binds the encoding path to the decoding path (cf. Fig. 1). It is a multiscale model with a large receptive field that can capture globally distributed artifacts^29^.

In detail, U-net input/output dimensions were set to 128 × 128 × 2. The two channels correspond to the real and imaginary parts of the input/output image, hence preserving the complex nature of the data. The encoding path involves a sequence of two convolution operations (size: 3 × 3, stride 1), followed by a ReLU activation function and max-pooling operation (size: 2 × 2, stride 2). This last step halves the spatial dimensions. This sequence is repeated four times and the number of filters doubles after each sequence. Similar to the encoding path, the decoding path consists of four sequences where each sequence involves an up-sampling operation to restore image size followed by two convolution operations. The decoding path additionally involves a concatenation operation, where a higher resolution feature map from the encoding path is concatenated to its corresponding feature map in the decoding path to recover details lost in the encoding path. The last layer is a convolution layer with 2 filters (size 1 × 1) used to map a 64-channel layer into real and imaginary outputs.

Lee et al*.* showed that a residual block can improve the reconstruction performance of a U-net model^35^. The residual block introduced in reference^36^ consisted of a shortcut connection that performs an identity map of U-net input and adds it to the output resulting in a ‘residual U-net’. Using the residual block instead of the original alias-free full image simplifies the topology structure which translates in easier and more accurate learning^35^. This method was used, later on, in many MR accelerated acquisition studies^10–12,14,37^ and is similarly employed in our work.

RMSProp was adopted as an optimizer with an adaptive learning rate starting from 10^–3^. The number of epochs was set arbitrarily to 2000. However, the model converged before reaching 2000 epochs without overfitting. The validation was done every epoch to avoid the potential overlooking of overfitting phenomena. Training and validation loss curves are shown in supplementary material (cf. supplementary Fig. S1). Additionally, repeatability experiments were carried out (cf. supplementary material). The pipeline was implemented in Python3 using the Keras library and TensorFlow^38^ as a backend. The learning computation was carried out on an Intel Xeon workstation associated with a Graphics Processing Unit (GPU) GeForce GTX 1080 Ti, and took a total time of 1.5 h.

### Conducted studies

The phase encoding directions (k_y_ and k_z_) were undersampled following 2D Gaussian sampling distributions, while a fixed number of *k*-space center lines (CL) was fully sampled to preserve low spatial frequency information and SNR. The study was divided into two parts: a retrospective analysis based on the results of two different experiments, and a prospective analysis performed on data acquired according to the retrospective outcomes.

#### Retrospective study

**Figure 2:**
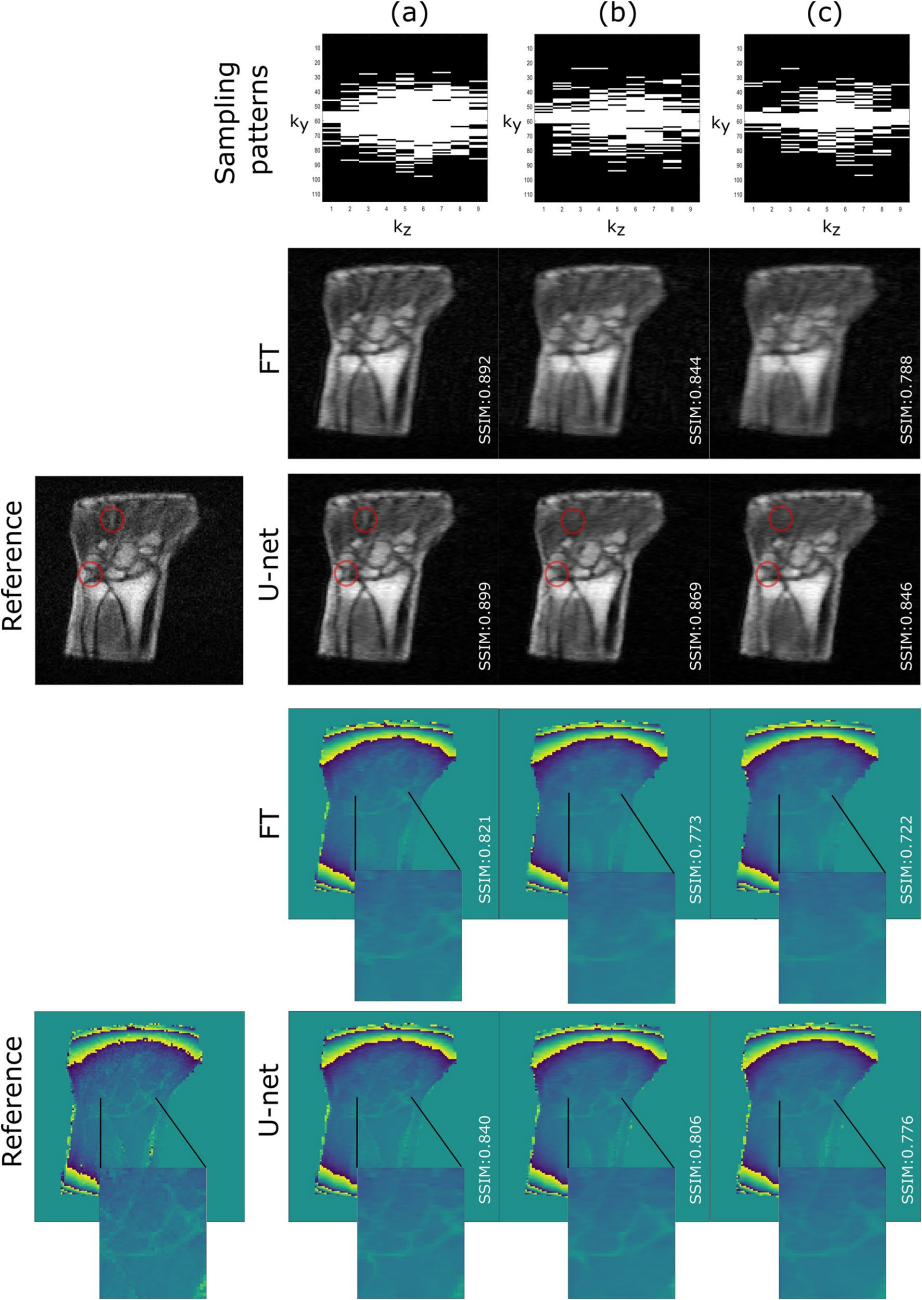
Model performance for different acceleration rates: (**a**) threefold, (**b**) fourfold and (**c**) fivefold. The red circles point out the missed details with higher acceleration rates in the magnitude images.

In a first experiment, the performance of the DL model was assessed for different acceleration rates. Fully sampled *k*-spaces were retrospectively undersampled using three Gaussian sampling patterns with the same number of CL = 7, the same variances ${\sigma }_{y}$ and ${\sigma }_{z}$ along the two-phase directions (${\sigma }_{y}$ = 0.10 and ${\sigma }_{z}$ = 0.20), but different acceleration rates R = 3, 4 and 5 (cf. Fig. 2, top row).

In a second experiment, the performance of residual U-net was investigated for the highest acceleration rate (R = 5) while changing the variance of the Gaussian sampling patterns, hence leading to various artifacts in the reconstructed images. Indeed, when a Gaussian sampling pattern is used, the lower ${\sigma }_{y}$ and ${\sigma }_{z}$, the closer to a low pass filter the sampling pattern, and the blurrier the resulting zero-filled images. Similar to noise artifacts, blurriness is globally distributed over the image. If ${\sigma }_{y}$ and ${\sigma }_{z}$ are high, high-frequencies are better preserved but at the cost of less-distributed or localized artifacts that we term ‘local’ artifacts. Here, four different sampling patterns were compared with fixed R = 5 and ${\sigma }_{z}$ = 0.2, and a) CL = 23 /${\sigma }_{y}$ = 0, b) CL = 7 $/{\sigma }_{y}$ = 0.10, c) CL = 7/ ${\sigma }_{y}$ = 0.15, and ultimately d) CL = 7/ ${\sigma }_{y}$ = 0.20 (cf. Fig. 4, top row).

#### Prospective study

A set of test data was acquired using a fivefold undersampled *k*-space acquisition (acquisition time ~ 3 min). An optimal sampling pattern was chosen according to the results of the previous experiment.

### Metrics

Most commonly accepted metrics in the field of image reconstruction were used to evaluate the model performance, namely Peak SNR (PSNR), structure similarity index (SSIM)^39^, and normalized root MSE (NRMSE).

- PSNR represents the ratio between the maximum intensity across a reference image $f$ and the MSE of the DL reconstructed image $\widehat{f}$:
  3 $$PSNR=20{\mathit{log}}_{10}\frac{max(f)}{\sqrt{\frac{1}{\mathit{M}}\sum_{\mathrm{i}=1}^{\mathit{M}}{(f\left(\mathrm{i}\right)- \widehat{f}(\mathrm{i}))}^{2}}}$$
  where M is the number of pixels in the image.
- The SSIM index attempts to quantify the perceptual differences between two images. The comparison is based on three features: luminance, contrast and structure. These features are evaluated at different image locations by using a sliding window. The resulting SSIM between two image patches A and B is given by:
  4 $$SSIM\left(A, B\right)= \frac{(2{\mu }_{A}{\mu }_{B}+{C}_{1})({\sigma }_{AB}+{C}_{2})}{({\mu }_{A}^{2}+{\mu }_{B}^{2}+{C}_{1})({\sigma }_{A}^{2}+{\sigma }_{B}^{2}+{C}_{2})}$$
  $\mu$ and $\sigma$ are the mean and the variance values of an image patch. ${C}_{1}$ and ${C}_{2}$ are two constants to stabilize the division. The reported SSIM in the rest of the document is the mean SSIM calculated as follows: $\frac{1}{P}\sum_{i=1}^{P}SSIM$, where $P$ is the total number of patches.
- NRMSE is the normalized root mean squared error. When normalized by the mean value of the reference image $f$, NRMSE is given by:
  5 $$NRMSE= \frac{\sqrt{\frac{1}{M}\sum_{i=1}^{M}{(f\left(i\right)- \widehat{f}(i))}^{2}}}{\mathit{mean}\left(f\right)}$$

Additionally, to assess the sharpness of the reconstructed images, 2D kernels for edge detection were employed in vertical and horizontal directions (${G}_{x}$ and ${G}_{y}$). The gradient magnitude $G$ is given in Eq. 6 and as opposed to the above metrics, *G* is a reference-free metric.

6 $$G=\mathit{mean}\left(\sqrt{{G}_{x}^{2}+{G}_{y}^{2}}\right)$$

This metric was only considered on magnitude images. In fact, in phase-contrast images, the contribution of phase wraps dominates compared to that of image details in the gradient calculation, translating in very challenging data interpretation. In general, considering that the phase is encoded between -pi and pi, we believe that only NRMSE and SSIM are appropriate metrics for phase images and were chosen as such in all further analyses.

A binary mask defined as a Region-of-Interest (ROI) for each image was generated. More specifically, a preliminary mask was first created based on pixels thresholding of the magnitude of the reference image. Then, a closing operation followed by an opening operation^40^, which are mathematical morphological operations, were performed in order to extract the ROI and remove the isolated noisy pixels. The resulting mask was used such that the background contribution (i.e., noise in the image) was excluded when computing all reconstruction metrics. Indeed, U-net generally leads to reconstructed images with a noticeably lower background noise than the reference images. This naturally leads to an underestimation of the quality of the reconstructed images when the entire field-of-view is considered.

Images of the 3D datasets that did not contain signal (i.e., noise only) were not included in the calculation of our reconstruction metrics.

Finally, to determine differences between groups or methods, non-parametric tests were carried out for each metric (Kruskal–Wallis and Wilcoxon), where a *p*-value < 0.05 was in general considered statistically significant (when relevant, p-values were adjusted using the Bonferroni correction to account for multiple comparisons).

## Results

### Retrospective study

**Figure 3:**
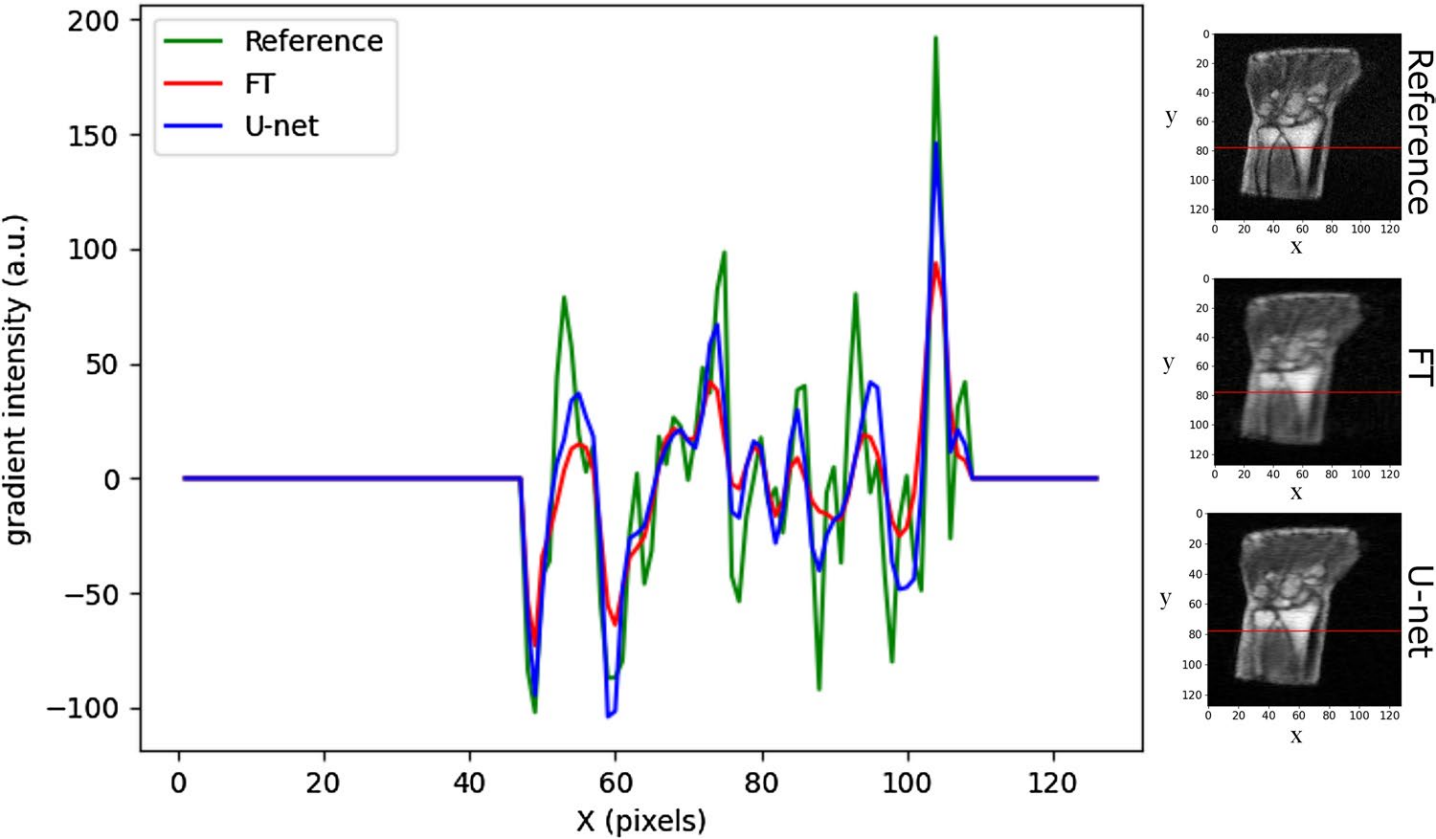
Example illustrating the model performance in preserving edges. Gradient profiles along the red line in reference (green), FT (red) and DL (blue) magnitude images with a fivefold acceleration rate (CL = 7, ${\sigma }_{y}= 0.10$).

The model performance was first evaluated at different acceleration rates (3, 4 and 5) with a Gaussian sampling pattern acting as a low pass filter (CL = 7, ${ \sigma }_{y}$ = 0.10 and ${\sigma }_{z}$ = 0.20). An example of the corresponding reconstructions is shown in Fig. 2. Residual U-net was able to reduce the blurriness in zero-filled Fourier transformed magnitude images for the three acceleration rates and provided smooth magnitude reconstructed images with sharper boundaries as illustrated by the gradient intensity profile shown in Fig. 3. In particular, the gradient metric indicated that residual U-net could better recover image sharpness and preserve the structures edges especially at high acceleration rates (see Table 1). For instance, the sharpness of the magnitude images improved in average over the test set by almost 13% at R = 5. However, higher acceleration rate progressively resulted in lower image quality where high spatial-frequencies and hence small details were more likely to be missed in the reconstructed magnitude images (cf. red circles in Fig. 2). Regarding phase images, interestingly, residual U-net was able to preserve not only the image sharpness but also most edges that were smoothed out in zero-filled phase images especially for high acceleration rates (cf. Fig. 2). As for magnitude images, the model performance was lower with higher acceleration rates. Our observations are supported by the quantitative results calculated over test data and summarized in Table 1. The statistical analysis on phase and magnitude metrics showed that DL reconstruction is significantly better than FT reconstruction for all acceleration rates (p-values < 0.01667) (cf. Tables S3 and S4 in supplementary material).

**Table 1:** Summary of performance obtained on the magnitude and phase images for different acceleration rates.

**Magnitude**					
Acceleration rate	Reconstruction method	PSNR	SSIM	NRMSE	Gradient
Threefold	FT	28.032 $\pm$ 1.640	0.883 $\pm$ 0.016	0.170 $\pm$ 0.068	37.643 $\pm$ 4.934
	U-net	29.121 $\pm$ 0.829**	0.896 $\pm$ 0.016**	0.145 $\pm$ 0.023**	40.402 $\pm$ 5.375**
Fourfold	FT	25.655 $\pm$ 1.375	0.836 $\pm$ 0.018	0.221 $\pm$ 0.070	34.757 $\pm$ 4.486
	U-net	27.698 $\pm$ 0.852**	0.863 $\pm$ 0.020**	0.170 $\pm$ 0.018**	38.974 $\pm$ 5.630**
Fivefold	FT	24.424 $\pm$ 1.284	0.797 $\pm$ 0.018	0.253 $\pm$ 0.071	32.767 $\pm$ 4.049
	U-net	26.642 $\pm$ 0.843**	0.834 $\pm$ 0.021**	0.192 $\pm$ 0.024**	37.029 $\pm$ 4.891**

**Phase**					
Acceleration rate	Reconstruction method	PSNR	SSIM	NRMSE	
Threefold	FT	25.890 $\pm$ 2.606	0.796 $\pm$ 0.024	0.083 $\pm$ 0.040	
	U-net	26.527 $\pm$ 2.229**	0.808 $\pm$ 0.028**	0.079 $\pm$ 0.036*	
Fourfold	FT	24.339 $\pm$ 2.910	0.741 $\pm$ 0.034	0.095 $\pm$ 0.047	
	U-net	25.703 $\pm$ 2.057**	0.767 $\pm$ 0.034**	0.087 $\pm$ 0.036*	
Fivefold	FT	23.530 $\pm$ 2.682	0.704 $\pm$ 0.034	0.101 $\pm$ 0.048	
	U-net	24.855 $\pm$ 2.442**	0.729 $\pm$ 0.040**	0.097 $\pm$ 0.045	

The stars indicate when differences in metrics between U-net and FT are statistically significant (* for p < 0.01667; ** for p << 0.001; the comparison not showing statistical significance had p-value = 0.073 for the phase image).

**Figure 4:**
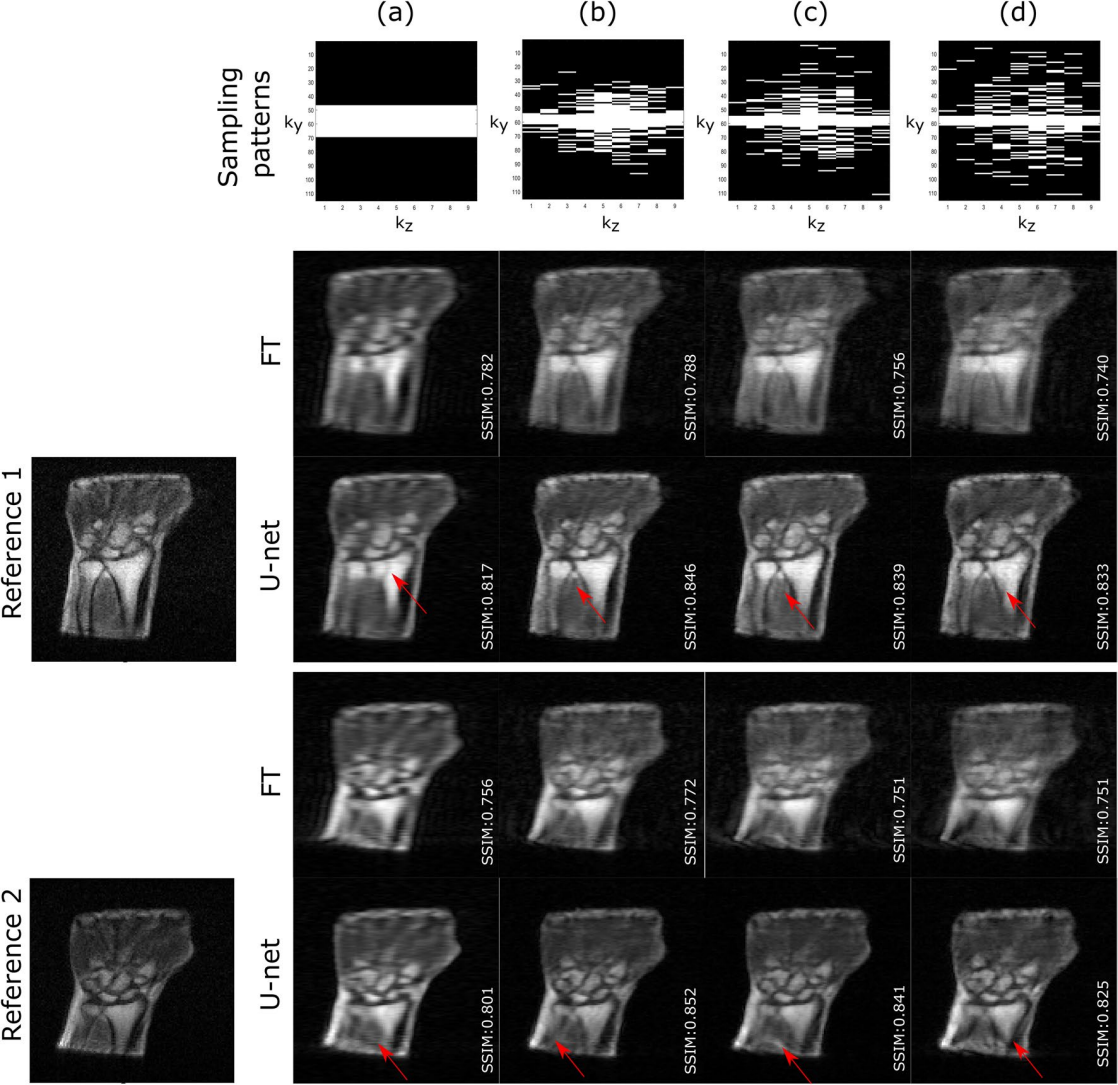
Impact of sampling patterns on the reconstruction performance of the magnitude images. (**a**) CL = 23 ${\sigma }_{y}$ = 0, (**b**) CL = 7 ${\sigma }_{y}$ = 0.10, (**c**) CL = 7 ${\sigma }_{y}$ = 0.15 and (**d**) CL = 7 ${\sigma }_{y}$ = 0.20. Acceleration rate = 5. Although the model succeeded in fully removing local artifacts in the background, red arrows show examples of local artifacts still present inside the ROIs.

**Figure 5:**
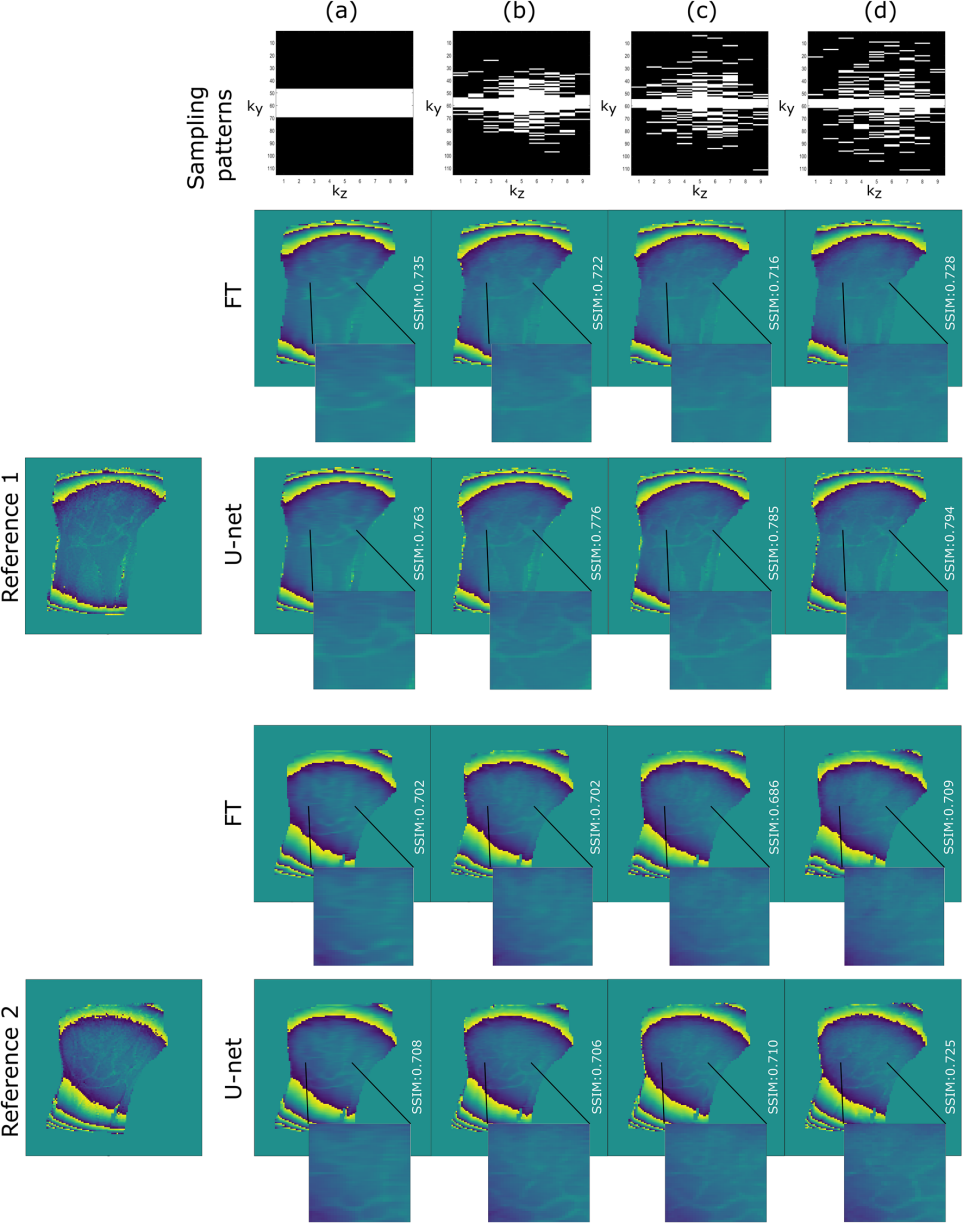
Impact of the sampling pattern on the reconstruction performance of the phase images. (**a**) CL = 23 ${\sigma }_{y}$ = 0, (**b**) CL = 7 ${\sigma }_{y}$ = 0.10, (**c**) CL = 7 ${\sigma }_{y}$ = 0.15 and (**d**) CL = 7 ${\sigma }_{y}$ = 0.20. Acceleration rate = 5.

The impact of different sampling patterns on the reconstruction performance at the highest acceleration rate R = 5 was also explored and the results are summarized in Table 2. Figures 4 and 5 show respectively magnitude and phase reconstruction examples corresponding to the four sampling patterns. Sampling pattern (a) led to highly blurred images after FT with local aliasing-type of artifacts in both the background and the ROIs. The proposed model enhanced the edges sharpness and corrected the artifacts in the background, yet could neither recover the lost higher frequencies nor correct for the local artifacts inside the ROI (cf. red arrows in Fig. 4). Sampling pattern (b) led to blurred Fourier transformed images with subtle, local artifacts. Therefore, the model reconstructed sharp and nearly artifact-free magnitude images. The non-centered sampling patterns (c) and (d), as expected, led to sharper zero-filled images. However, local artifacts were still visible in the ROIs depending on the MR images. Regarding phase images, larger ${\sigma }_{y}$ led to higher details recovery. The local artifacts seen on magnitude images are less obvious in phase images.

**Table 2:** Summary of performance obtained on magnitude and phase images for different sampling patterns.

**Magnitude**				
Sampling patterns	PSNR	SSIM	NRMSE	Gradient
CL = 7; ${\sigma }_{y}=$ 0	25.692 $\pm$ 1.317	0.814 $\pm$ 0.020	0.218 $\pm$ 0.062	35.113 $\pm$ 4.381
CL = 7; ${\sigma }_{y}=$ .10	26.642 $\pm$ 0.847	0.834 $\pm$ 0.021	0.192 $\pm$ 0.024	37.029 $\pm$ 4.891
CL = 7; ${\sigma }_{y}=$.15	25.998 $\pm$ 1.081	0.822 $\pm$ 0.027	0.206 $\pm$ 0.023	37.710 $\pm$ 5.544
CL = 7; ${\sigma }_{y}=$ .20	25.961 $\pm$ 0.765	0.814 $\pm$ 0.024	0.208 $\pm$ 0.034	36.387 $\pm$ 4.666

**Phase**				
Sampling patterns	PSNR	SSIM	NRMSE	
CL = 7; ${\sigma }_{y}=$ 0	24.700 $\pm$ 2.751	0.721 $\pm$ 0.029	0.094 $\pm$ 0.044	
CL = 7; ${\sigma }_{y}=$ .10	24.855 $\pm$ 2.442	0.728 $\pm$ 0.040	0.096 $\pm$ 0.045	
CL = 7; ${\sigma }_{y}=$.15	25.046 $\pm$ 2.518	0.734 $\pm$ 0.048	0.100 $\pm$ 0.047	
CL = 7; ${\sigma }_{y}=$ .20	25.961 $\pm$ 0.765	0.729 $\pm$ 0.040	0.097 $\pm$ 0.046	

Acceleration rate = 5.

Apart from sampling pattern (a) or ${\sigma }_{y}=0$ which translated in the lowest performance, the rest of the sampling patterns show, overall, similar quantitative results. Nevertheless, the sampling pattern (b) or ${\sigma }_{y}=0.10$ demonstrates improved PSNR, SSIM and NRMSE on magnitude images (cf. Tables S5 and S6 in supplementary material) and led to nearly artifact-free images of 3D LF wrist/hand test data. Therefore, this pattern was used to evaluate the performance of the model on prospectively acquired data.

### Prospective study

**Figure 6:**
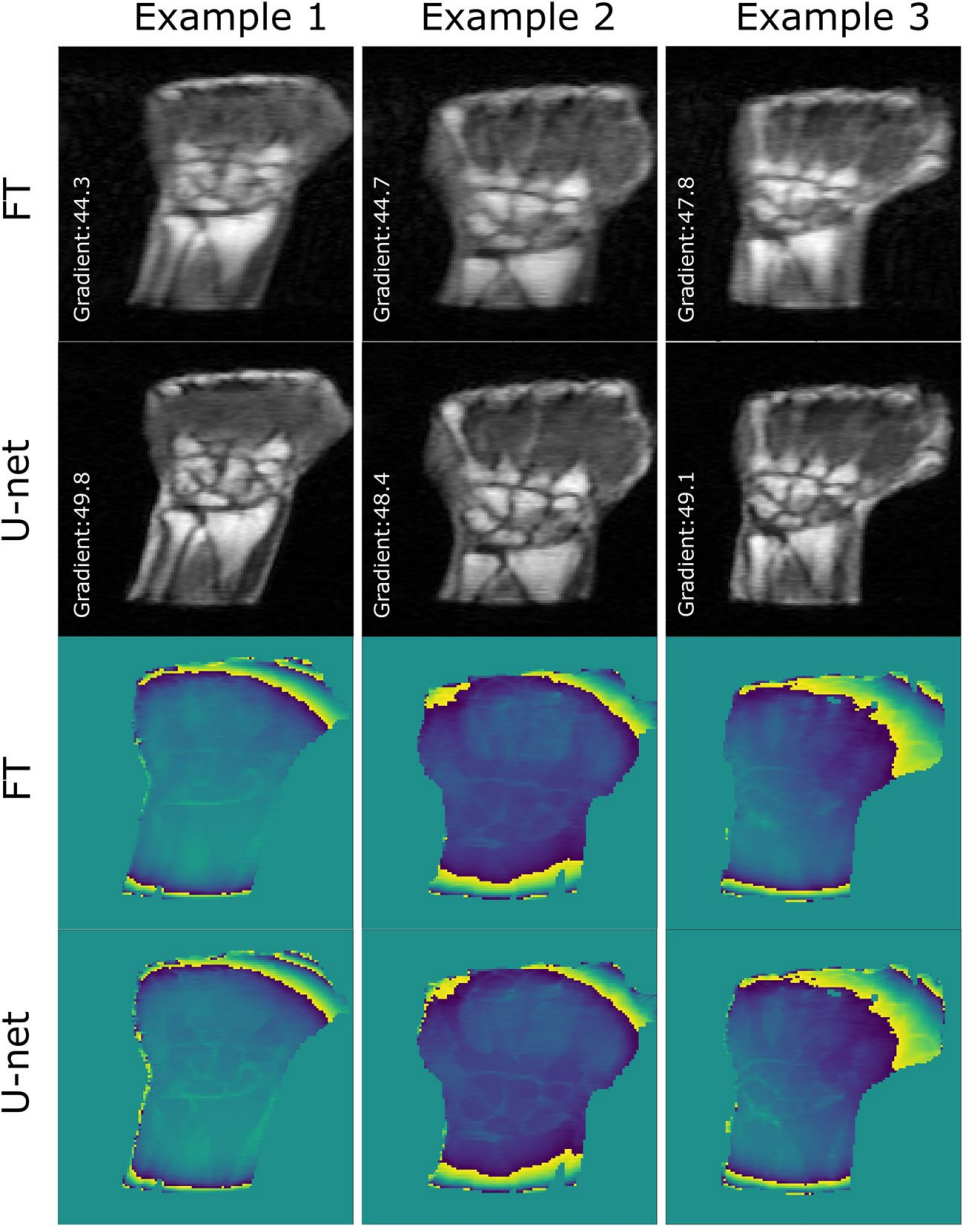
Reconstruction results of 3 different prospectively undersampled MR data with the Gaussian sampling pattern: CL = 7, ${\sigma }_{y}=0.10$. Acceleration rate = 5.

Figure 6 shows three examples of fivefold undersampled MR data. A similar behavior to retrospective undersampling was observed; namely, edges were sharper, and edges lost in the Fourier transformed phase images were recovered. Table 3 shows an increase in gradient values of 9.4% for magnitude images.

**Table 3:** Results of prospectively acquired MR images.

	Reconstruction method	Gradient
Magnitude images	FT	40.470 $\pm$ 3.355
	U-net	44.286 $\pm$ 3.307

## Discussion

In this work, we investigated the potential of residual U-net, a data-driven model, to reconstruct 3D LF undersampled MR images in combination with small databases. Data augmentation with basic image transformations was adopted to mitigate the small size of the training dataset. The performance of this approach for three different acceleration rates was first assessed on retrospectively undersampled LF data. Quantitative results showed a statistically significant improvement when U-net reconstruction was used instead of conventional iFT. More specifically, the proposed DL approach was able to recover the image sharpness, even for high acceleration rates. We believe that sharpness was improved as a consequence of a reduced global distributed artifact –blurriness in the present case—generated by the sampling pattern rather than an explicit learning of the anatomical edges or details. The global artifact correction was most likely the result of data augmentation coupled to the U-net architecture nature. Indeed, the model learned at each iteration a newly-generated undersampled real/imaginary data having the same global artifact and learned to correct for it without overfitting details of the image. Moreover, considering its large receptive field, U-net was often used to remove incoherent globally distributed artifacts such as noise or streaking artifacts^11,12,29,41,42^. Thus, the large receptive field of U-net proves once more to be suitable for correcting a global distributed artifact. Phase images were reconstructed with equal performance as magnitude images, thus opening new perspectives for MR techniques relying on phase-contrast imaging. The edges appearing smoothed in zero-filled FT phase-contrast images were sharper using DL reconstruction. One can think that this could be caused by an explicit anatomical learning. However, since this information was already present in the DL model inputs (i.e., the real and imaginary components of Fourier transformed image as illustrated in Fig. S4 in supplementary material), we hypothesize that it originates from a reduced global blurriness of real and imaginary components inherited from the *k*-space sampling pattern. Accordingly, in the context of pathological cases, we would assume that, as long as the pathology information is present in a “blurred” form in the DL model input, it can be restored without artifact. However, assuming that pathology would take the shape of very small structural changes lost from the undersampling scheme used, there could indeed be a risk that the model forces the reconstructions toward a healthy wrist appearance. That said, a solution could be to settle on a minimum resolution loss that would limit such a risk, to recover structures of a minimum size. Then of course, as for any imaging modality and regardless of DL reconstruction pipelines, a general lack of resolution may prevent to recognize small pathological structures that would be lost from partial volume effects. In the latter case though, DL is not the limiting factor.

One limitation of our DL reconstruction comes from some remaining smoothness in both magnitude and phase images though. This could be due to 1- the undersampling patterns inevitably missing high spatial frequencies in *k*-space, and/or 2- the MSE loss function that tends to reduce the noise over flat intensity regions, translating in noiseless textures on the reconstructed image^43^. For the employed sampling pattern (CL = 7, ${\sigma }_{y}$ = 0.10), this loss of higher spatial frequencies is further exacerbated by the higher acceleration rate (fivefold), both in the U-net reconstructed magnitude and phase images, as illustrated by the lower metrics shown in Table 1.

In line with this hypothesis, an attempt to maintain the high spatial frequencies in the reconstructed images was conducted by investigating different sampling patterns (${\sigma }_{y}$ values 0, 0.10, 0.15 and 0.20) for R = 5. As expected, higher ${\sigma }_{y}$ led to better details preservation in both reconstructed magnitude and phase images. However, U-net was not able to correct for the local artifacts generated by the different sampling patterns, especially inside the ROIs of the magnitude images. These generated artifacts are highly dependent on the sampling pattern when dealing with small learning dataset. This in turn means that the proposed approach cannot be easily generalized. Additional concerns on model generalization arise from the homogenous sequence parameters that we deliberately used so far for the training set. Indeed, as the knowledge in this specific field of research is still in its infancy, we believe it is important to limit the degrees of freedom that might influence the model performance. In future work, we will explore different solutions for enhanced flexibility by using sampling patterns less prone to local artifacts such as variable density Poisson-disc. And, for clinical relevance, we will study the generalization ability of our model with respect to different sequence parameters.

Additional improvement can be obtained using other types of loss functions. Although we employed in this work one of the most commonly used MSE, future work includes exploring the use of perceptual loss to improve the reconstruction performance^44,45^.

Finally, as opposed to most undersampled MR studies using DL, we prospectively tested our trained model on undersampled wrist MR images using the highest acceleration rate (fivefold) and ${\sigma }_{y}$ = 0.10. The results on magnitude and phase images confirmed that our model performs similarly on both simulated and acquired data. This key aspect has two major advantages. First, our method could be directly used in concrete applications to reconstruct full MR complex data without further optimization steps. Second, it could also be used to fine-tune with confidence the sampling pattern on simulated data without the need of collecting extra data.

As future steps, further investigations will be carried out to compare the effectiveness of simulated data against data augmentation, as well as to assess the potential added value of transfer learning approach^46^ based on natural or MR datasets compared to the present work. Ultimately, different promising network architectures will be considered in future work such as model-driven^5^ methods (unrolled optimization algorithms) and transformers^47^.

## Conclusion

In this work, we demonstrated the potential of residual U-net for accelerating LF 3D MRI acquisitions. Promising results were obtained not only on magnitude but also on phase reconstructed images for three to fivefold acceleration rates. Although a relatively small (n = 10) dataset was used for training, data augmentation based on geometric image manipulations successfully mitigated data scarcity and allowed complex-valued MR data recovery. The global structure and image sharpness were preserved in the reconstructed magnitude and phase images. Additionally, our results indicated that the model performances are tied to the adopted sampling pattern. Nevertheless, promising results were obtained not only on simulated data but also on acquired fivefold accelerated MR data. This highlights the substantial potential of emerging DL approaches to significantly speed up LF MR acquisitions and ultimately to bring LF MR closer to clinical applications.

## Supplementary Information

Supplementary Information.

## Data Availability

The data that support the findings of this study are available on request from the corresponding author (R. Ayde) for noncommercial, research purposes.

## References

[1] Sarracanie M, Salameh N (2020). Low-field MRI: How low can we go? A fresh view on an old debate. Front. Phys..

[2] Wald LL (2019). Ultimate MRI. J. Magn. Reson..

[3] Klein, H.-M. *Clinical Low Field Strength Magnetic Resonance Imaging*. (Springer International Publishing, 2016). 10.1007/978-3-319-16516-5.

[4] Schukro C, Puchner SB (2019). Safety and efficiency of low-field magnetic resonance imaging in patients with cardiac rhythm management devices. Eur. J. Radiol..

[5] Liang D, Cheng J, Ke Z, Ying L (2020). Deep MRI reconstruction: Unrolled optimization algorithms meet neural networks. IEEE Sig. Proc. Mag..

[6] Lustig M, Donoho DL, Santos JM, Pauly JM (2008). Compressed sensing MRI. IEEE Signal Process. Mag..

[7] Pruessmann KP, Weiger M, Scheidegger MB, Boesiger P (1999). SENSE: Sensitivity encoding for fast MRI. Magn. Reson. Med..

[8] Bernstein, M. A., King, K. F. & Zhou, X. J. *Handbook of MRI pulse sequences*. (Elsevier Academic Press, 2004).

[9] Darrasse L (2003). Perspectives with cryogenic RF probes in biomedical MRI. Biochimie.

[10] Schlemper, J., Caballero, J., Hajnal, J. V., Price, A. & Rueckert, D. A deep cascade of convolutional neural networks for dynamic MR image reconstruction. arXiv:1704.02422 [cs] (2017). 10.1109/TMI.2017.2760978 29035212

[11] Souza, R. & Frayne, R. A hybrid frequency-domain/image-domain deep network for magnetic resonance image reconstruction. arXiv:1810.12473 [cs, eess, stat] (2018).

[12] Souza, R. *et al.* Dual-domain cascade of U-nets for multi-channel magnetic resonance image reconstruction. arXiv:1911.01458 [physics, stat] (2019). 10.1016/j.mri.2020.06.002 32562744

[13] Zhu B, Liu JZ, Cauley SF, Rosen BR, Rosen MS (2018). Image reconstruction by domain-transform manifold learning. Nature.

[14] Yang G (2018). DAGAN: deep de-aliasing generative adversarial networks for fast compressed sensing MRI reconstruction. IEEE Trans. Med. Imaging.

[15] Hammernik K (2018). Learning a variational network for reconstruction of accelerated MRI data: Learning a variational network for reconstruction of accelerated MRI data. Magn. Reson. Med.

[16] Menze BH (2015). The multimodal brain tumor image segmentation benchmark (BRATS). IEEE Trans. Med. Imaging.

[17] Yan K, Wang X, Lu L, Summers RM (2018). DeepLesion: Automated mining of large-scale lesion annotations and universal lesion detection with deep learning. J. Med. Imag..

[18] Mueller SG (2005). The Alzheimer’s disease neuroimaging initiative. Neuroimaging Clin. N. Am..

[19] Essen, D. *et al.* The WU-Minn human connectome project: An overview. *J. NeuroImage* 38 (2014). 10.1016/j.neuroimage.2013.05.041 PMC3724347 23684880

[20] Zbontar, J. *et al.* fastMRI: An open dataset and benchmarks for accelerated MRI. arXiv:1811.08839 [physics, stat] (2019).

[21] Nath, R., Callahan, S., Singam, N., Stoddard, M. & Amini, A. A. accelerated phase contrast magnetic resonance imaging via deep learning. in *2020 IEEE 17th International Symposium on Biomedical Imaging (ISBI)* 834–838 (IEEE, 2020). 10.1109/ISBI45749.2020.9098508.

[22] Lee, D., Yoo, J., Tak, S. & Ye, J. C. Deep residual learning for accelerated MRI using magnitude and phase networks. arXiv:1804.00432 [cs, stat] (2018). 10.1109/TBME.2018.2821699 29993390

[23] Schlemper, J. *et al.* Deep learning MRI reconstruction in application to point-of-care MRI. *ISMRM* (2020).

[24] Koonjoo, N., Zhu, B., Bagnall, C. & Rosen, M. *Boosting the signal-to-noise of low-field MRI&nbsp; With deep learning image reconstruction*. https://www.researchsquare.com/article/rs-126917/v1 (2020). 10.21203/rs.3.rs-126917/v1. 10.1038/s41598-021-87482-7 PMC8050246 33859218

[25] Knoll F (2019). Assessment of the generalization of learned image reconstruction and the potential for transfer learning. Magn. Reson. Med.

[26] Shorten C, Khoshgoftaar TM (2019). A survey on image data augmentation for deep learning. J. Big Data.

[27] Ronneberger, O., Fischer, P. & Brox, T. U-Net: Convolutional networks for biomedical image segmentation. arXiv:1505.04597 [cs] (2015).

[28] Hyun CM, Kim HP, Lee SM, Lee S, Seo JK (2018). Deep learning for undersampled MRI reconstruction. Phys. Med. Biol..

[29] Han, Y. S., Yoo, J. & Ye, J. C. Deep residual learning for compressed sensing CT reconstruction via persistent homology analysis. arXiv:1611.06391 [cs] (2016).

[30] Han, Y. S., Yoo, J. & Ye, J. C. Deep learning with domain adaptation for accelerated projection-reconstruction MR. arXiv:1703.01135 [cs] (2018). 10.1002/mrm.27106 29399869

[31] Constantinesco A (1997). Low-field dedicated and desktop magnetic resonance imaging systems for agricultural and food applications. Magn. Reson. Chem..

[32] Sarracanie, M. Fast quantitative low-field magnetic resonance imaging with OPTIMUM—optimized magnetic resonance fingerprinting using a stationary steady-state cartesian approach and accelerated acquisition schedules. *Investig. Radiol. Publish *Ahead of Print (2021). 10.1097/RLI.0000000000000836 PMC8903217 34669651

[33] Chollet, F. keras: https://github.com/fchollet/keras. (2015).

[34] Eldele, E. eldeen. Tweaked Image Generator: https://gist.github.com/Emadeldeen24/736c33ac2af0c00cc48810ad62e1f54a. (2019).

[35] Lee, D., Yoo, J. & Ye, J. C. Deep residual learning for compressed sensing MRI. in *2017 IEEE 14th International Symposium on Biomedical Imaging (ISBI 2017)* 15–18 (IEEE, 2017). 10.1109/ISBI.2017.7950457.

[36] He, K., Zhang, X., Ren, S. & Sun, J. Deep Residual learning for image recognition. in *2016 IEEE Conference on Computer Vision and Pattern Recognition (CVPR)* 770–778 (IEEE, 2016). 10.1109/CVPR.2016.90.

[37] Eo T (2018). KIKI-net: Cross-domain convolutional neural networks for reconstructing undersampled magnetic resonance images. Magn. Reson. Med..

[38] Abadi, M. *et al.* TensorFlow: Large-scale machine learning on heterogeneous distributed systems. arXiv:1603.04467 [cs] (2016).

[39] Wang Z, Bovik AC, Sheikh HR, Simoncelli EP (2004). Image quality assessment: From error visibility to structural similarity. IEEE Trans. Image Process..

[40] Jamil, N., Sembok, T. M. T. & Bakar, Z. A. Noise removal and enhancement of binary images using morphological operations. in *2008 International Symposium on Information Technology* 1–6 (IEEE, 2008). 10.1109/ITSIM.2008.4631954.

[41] Tamada, D. Review: Noise and artifact reduction for MRI using deep learning. arXiv:2002.12889 [physics] (2020).

[42] Jin KH, McCann MT, Froustey E, Unser M (2017). Deep convolutional neural network for inverse problems in imaging. IEEE Trans. Image Process..

[43] Zhao, H., Gallo, O., Frosio, I. & Kautz, J. loss functions for neural networks for image processing. arXiv:1511.08861 [cs] (2018).

[44] Johnson, J., Alahi, A. & Fei-Fei, L. Perceptual losses for real-time style transfer and super-resolution. in *Computer Vision – ECCV 2016* (eds. Leibe, B., Matas, J., Sebe, N. & Welling, M.) vol. 9906 694–711 (Springer International Publishing, 2016).

[45] Ghodrati V (2019). MR image reconstruction using deep learning: Evaluation of network structure and loss functions. Quant. Imaging Med. Surg..

[46] Dar, S. U. H., Özbey, M., Çatlı, A. B. & Çukur, T. A Transfer‐learning approach for accelerated MRI using deep neural networks. *Magn. Reson. Med.* 23 (2020). 10.1002/mrm.28148. 10.1002/mrm.28148 31898840

[47] Guo, P., Mei, Y., Zhou, J., Jiang, S. & Patel, V. M. ReconFormer: Accelerated MRI reconstruction using recurrent transformer. arXiv:2201.09376 [cs, eess] (2022). 10.1109/TMI.2023.3314747 PMC10764001 37703139

