# Supplementary material for: Deep learning for fast low-field MRI acquisitions
Source: Sci Rep. 2022 Jul 6;12:11394. doi: 10.1038/s41598-022-14039-7 (PMC9259619; doi:10.1038/s41598-022-14039-7)
Supplement: Supplementary file 1 — Supplementary Information. [file 41598_2022_14039_MOESM1_ESM.docx]

**Deep learning for fast low field MRI acquisitions**

Reina AYDE, Tobias SENFT, Najat SALAMEH, Mathieu SARRACANIE

**Supplementary material**

***Training details***

**Supplementary figure 1:** The training (blue) and validation (orange) curves of the all models employed in the paper. These curves represent the evolution of the mean squared error (MSE) of the training/validation set with the number of epochs. Training batch size was set to 1 and validation was done every epoch.

***Repeatability***

Repeatability of model training is a key matter for DL applications. In our context, we can investigate it from two main standpoints. First, according to Alahmari *et al.*^1^ and assuming the same software and hardware settings, uncontrolled software randomization during the learning process (such as atomic addition operations and reduction approximation during back-propagation) may lead to nonrepeatable results. Although there exist ways to control randomization, these do not apply to our TensorFlow version (v1.5), which infers possible sources of variability when repeating the training. To assess the latter, we repeated the training of the reconstruction model (sampling pattern: CL=7 $\sigma_{y}$=0.10) 4 times. An example of the reconstructed images obtained with the different models is shown in supplementary figure 2. Additionally, the quantitative metrics were calculated over the test set for each model and a Kruskal-Wallis test was performed for each metric (cf. supplementary table 1). The results show no significant difference between the different repetitions, both qualitatively and quantitatively.


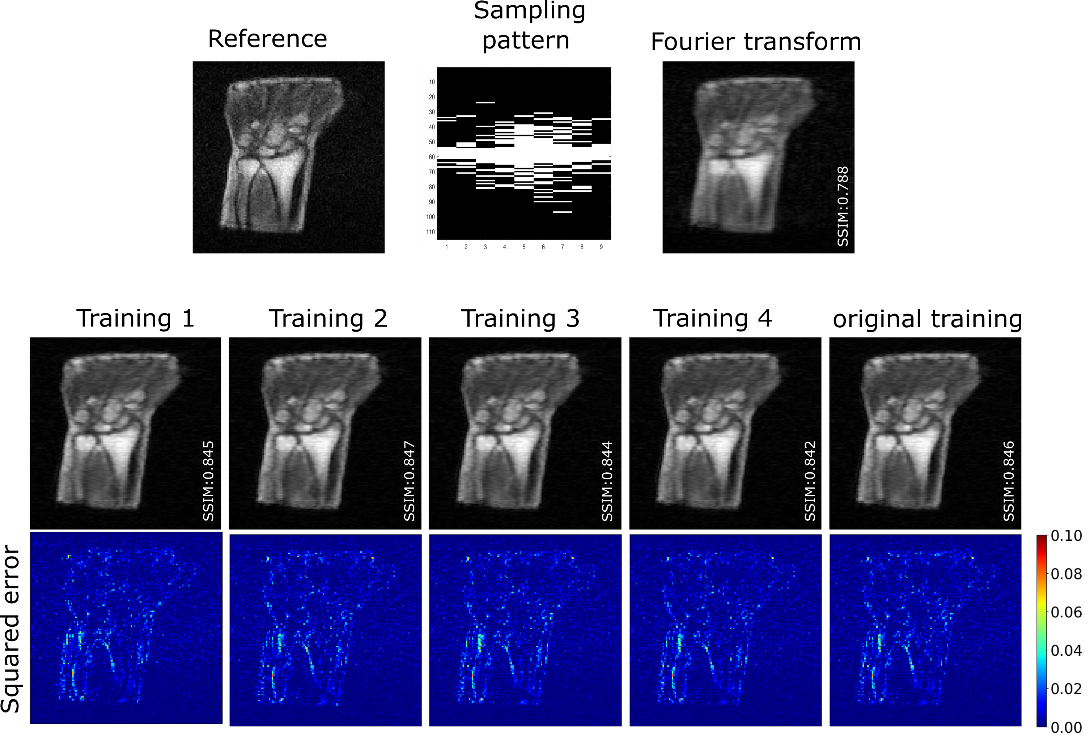


**Supplementary figure 2:** An example of model performance for different training repetitions (all training parameters were kept the same).

**Supplementary table 1:** p-values of Kruskal-Wallis tests on the 5 repeated trainings. The table shows no significant differences between the resulting models, neither on magnitude nor on phase reconstructed images.

|  |  | **SSIM** | **PSNR** | **NRMSE** | **Gradient** |
| --- | --- | --- | --- | --- | --- |
| **p-values** | **Magnitude** | 1.000 | 0.998 | 1.000 | 0.994 |
|  | **Phase** | 1.000 | 0.994 | 1.000 |  |

The second source of variability lies in the dataset. Splitting the latter into training and validation sets is usually done randomly which might lead to unintentional biased data selection. To assess potential variability induced by the dataset, 5 different trainings (sampling pattern: CL=7 $\sigma_{y}$=0.10) were carried out where training/validation sets were varied according to the following schemes:

| **Trainings #** | **Training/validation sets indexes** |
| --- | --- |
| Training 1 | [1:8]/[9:10] |
| Training 2 | [1:6][9:10]/[7:8] |
| Training 3 | [1:4][7:10]/[5:6] |
| Training 4 | [1:2][5:10]/[3:4] |
| Training 5 | [3:10]/[1:2] |

The same qualitative and quantitative analysis as described above were carried out (cf. supplementary figure 3 and table 2). The results show a non-significant difference between the different repetitions. Consequently, we believe that our experiments’ repeatability is good.


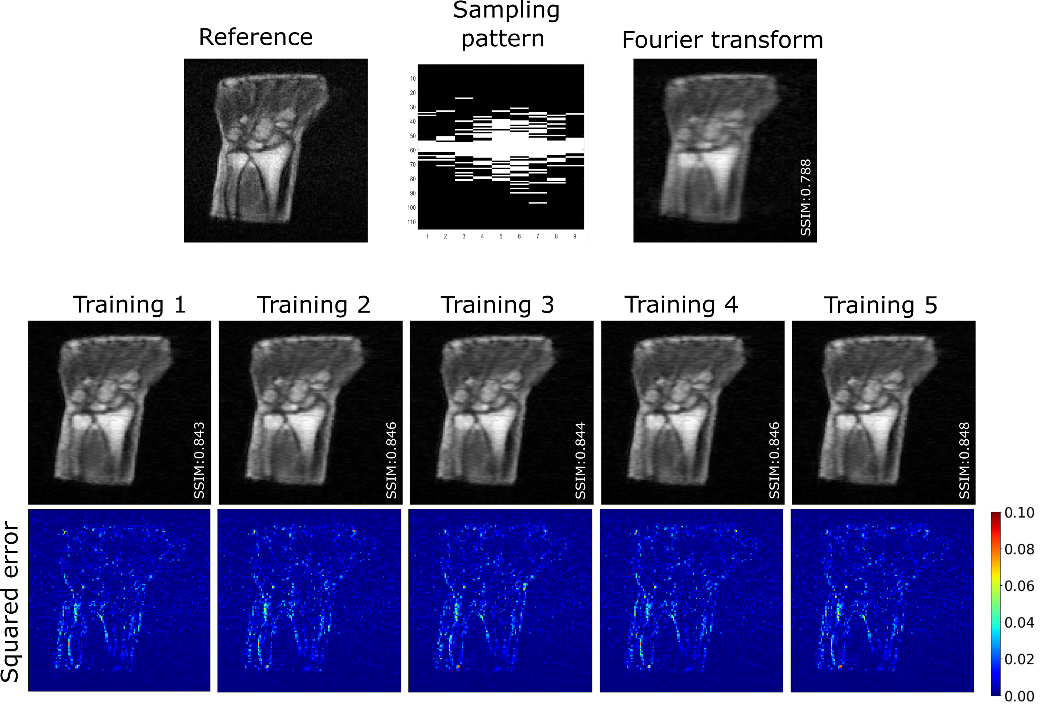


**Supplementary figure 3:** An example of model performance for different trainings where the training and validation datasets were varied.

**Supplementary table 2:** p-values of Kruskal-Wallis tests on the 5 trainings where the training and validation datasets were varied. The table shows no significant differences between the resulting models, neither on magnitude nor on phase reconstructed images.

|  |  | **SSIM** | **PSNR** | **NRMSE** | **Gradient** |
| --- | --- | --- | --- | --- | --- |
| **p-values** | **Magnitude** | 0.999 | 0.991 | 0.998 | 1.000 |
|  | **Phase** | 1.000 | 0.999 | 1.000 |  |

***Statistical analysis***

| **Magnitude** | | | | |
| --- | --- | --- | --- | --- |
| **Sampling rates** | **PSNR** | **SSIM** | **NRMSE** | **Gradient** |
| **3-fold** | 0.000197 | 0.000630 | 0.000197 | 0.001847 |
| **4-fold** | 0.000197 | 0.000197 | 0.000197 | 0.000536 |
| **5-fold** | 0.000197 | 0.000197 | 0.000197 | 0.000328 |

**Supplementary table 3**: p-values from the 2-by-2 comparison (Wilcoxon) between U-net and FT magnitude reconstruction. The table shows a general improvement for all sampling rates and all the metrics (p-values < 0.01667 are considered statistically significant).

| **Phase** | | | |
| --- | --- | --- | --- |
| **Sampling rate** | **PSNR** | **SSIM** | **NRMSE** |
| **3-fold** | 0.000864 | 0.000456 | 0.003286 |
| **4-fold** | 0.000277 | 0.000234 | 0.002472 |
| **5-fold** | 0.000536 | 0.000738 | 0.070710 |

**Supplementary table 4**: p-values from the 2-by-2 comparison (Wilcoxon) between U-net and FT phase-contrast reconstruction. The table shows a general improvement for all sampling rates and all the metrics (p-values < 0.01667 are considered statistically significant).

| **Magnitude** | | | | |
| --- | --- | --- | --- | --- |
| **Sampling patterns** | **PSNR** | **SSIM** | **NRMSE** | **Gradient** |
| $\boldsymbol{\sigma}_{\mathbf{y}}$**=0.10 –**$\boldsymbol{\sigma}_{\mathbf{y}}$**=** **0.15** | 0.000197 | 0.000197 | 0.000234 | 0.000234 |
| $\boldsymbol{\sigma}_{\mathbf{y}}$**=0.10 –**$\boldsymbol{\sigma}_{\mathbf{y}}$**=** **0.20** | 0.002139 | 0.000197 | 0.002853 | 0.170115 |
| $\boldsymbol{\sigma}_{\mathbf{y}}$**=0.15 –**$\boldsymbol{\sigma}_{\mathbf{y}}$**=** **0.20** | 0.001847 | 0.000328 | 0.000456 | 0.000277 |

**Supplementary table 5:** p-values from the 2-by-2 comparison (Wilcoxon) on magnitude reconstructed images between different sampling patterns ($\sigma_{y}$= 0.10, 0.15 and 0.20; p-values < 0.025 are considered statistically significant).

| **Phase** | | | |
| --- | --- | --- | --- |
| **Sampling patterns** | **PSNR** | **SSIM** | **NRMSE** |
| $\boldsymbol{\sigma}_{\mathbf{y}}$**=0.10 –**$\boldsymbol{\sigma}_{\mathbf{y}}$**=** **0.15** | 0.947916 | 0.038579 | 0.982628 |
| $\boldsymbol{\sigma}_{\mathbf{y}}$**=0.10 –**$\boldsymbol{\sigma}_{\mathbf{y}}$**=** **0.20** | 0.810698 | 0.711251 | 0.093604 |
| $\boldsymbol{\sigma}_{\mathbf{y}}$**=0.15 –**$\boldsymbol{\sigma}_{\mathbf{y}}$**=** **0.20** | 0.982628 | 0.034671 | 0.472400 |

**Supplementary table 6:** p-values from the 2-by-2 comparison (Wilcoxon) on phase-contrast reconstructed images between different sampling patterns ($\sigma_{y}$= 0.10, 0.15 and 0.20; p-values < 0.025 are considered statistically significant).


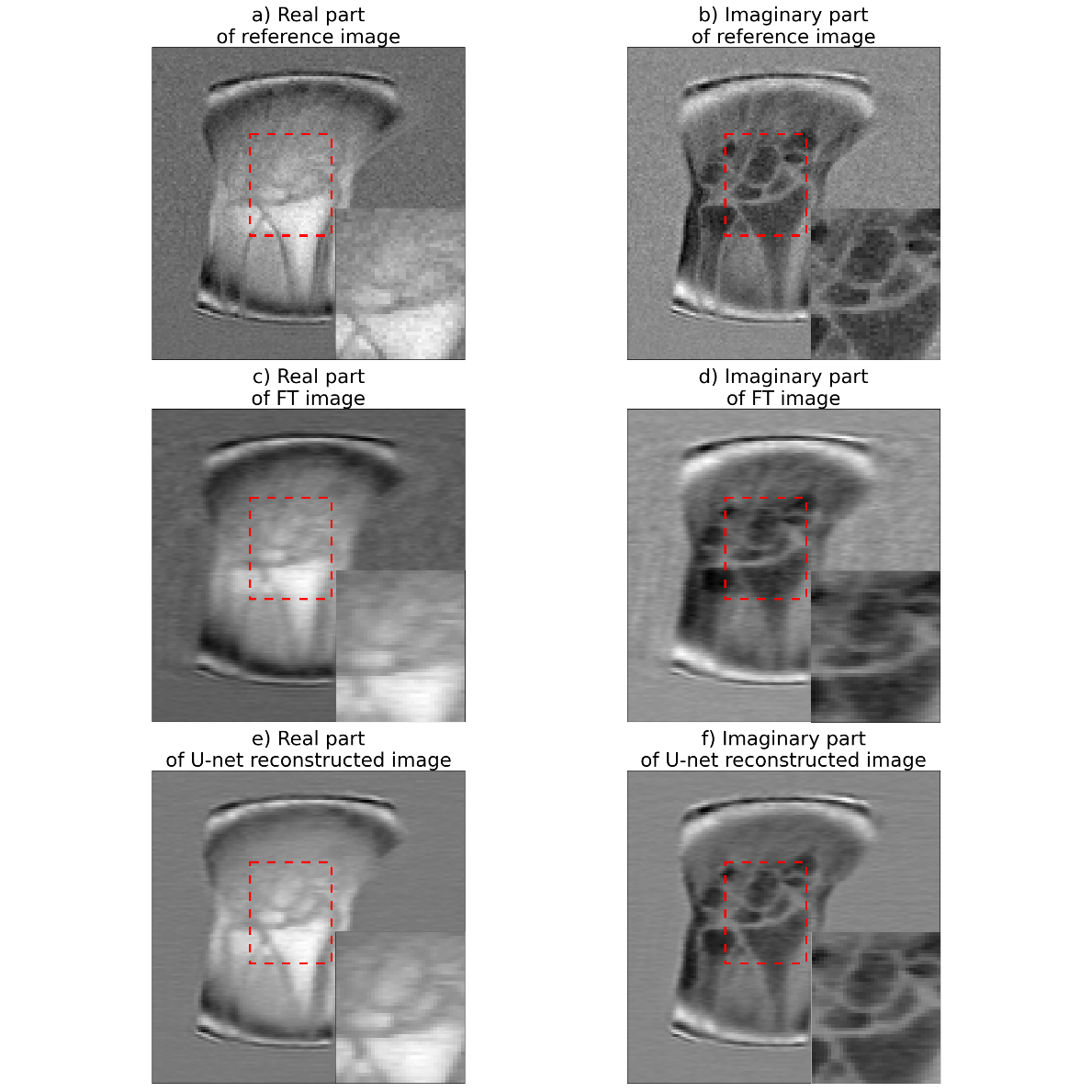


**Supplementary figure 4:** Real and imaginary parts of reference (a-b), FT- (c-d) and DL-reconstructed images (e-f) with 5-fold acceleration rate (${CL=7, \sigma}_{y}= 0.10$). The apparently lost edges in phase-contrast images (cf. Figure 2, R = 5) are still present in the real and imaginary components of FT image (c and d).

**References**

1. Alahmari, S. S., Goldgof, D. B., Mouton, P. R. & Hall, L. O. Challenges for the Repeatability of Deep Learning Models. *IEEE Access* **8**, 211860–211868 (2020).
